# *bolA* gene involved in curli amyloids and fimbriae production in *E. coli*: exploring pathways to inhibit biofilm and amyloid formation

**DOI:** 10.1186/s40709-020-00120-7

**Published:** 2020-06-17

**Authors:** Mohd W. Azam, Azna Zuberi, Asad U. Khan

**Affiliations:** 1 Interdisciplinary Biotechnology Unit, Aligarh Muslim University, Aligarh, UP 202002 India; 2 Medical Microbiology and Molecular Biology Lab, Interdisciplinary Biotechnology Unit, Aligarh Muslim University, Aligarh, 202002 India

**Keywords:** *Escherichia coli*, Biofilm, *bolA*, CRISPRi, dCas9, Extra polymeric substance (EPS), *bolA* gene knockdown strain (*bol*-KD)

## Abstract

**Background:**

Biofilm formation is a complex phenomenon of bacterial cells, involved in several human infections. Its formation is regulated and controlled by several protein factors. The BolA-like proteins (*bolA* gene) are conserved in both prokaryotes and eukaryotes. The BolA protein is a transcription factor involved in bacterial cell motility and biofilm formation. This study was initiated to elucidate the role of the *bolA* gene in the curli biogenesis and amyloid production as well as to observe changes in the expression of *fimH*, a fimbriae gene.

**Methods:**

Knockdown mutants of *Escherichia coli* MG1655 *bolA* gene *(bolA*-*KD)* were generated using CRISPR interference. The results obtained, were validated through gene expression using RT-PCR, microscopic analysis and different biofilm and amyloid assays.

**Results:**

The *bolA* knockdown mutants showed a decrement in curli amyloid fibers, in fimbriae production and biofilm formation. We have also observed a reduction in EPS formation, eDNA production and extracellular protein content. Gene expression data showed that *bolA* downregulation caused the suppression of *csgA* and *csgD* of curli that led to the reduction in curli fiber and the amyloid formation and also the suppression of *fimH*, leading to the loss of fimbriae.

**Conclusions:**

Curli fibers and fimbriae are found to be involved in biofilm formation leading to the pathogenicity of the bacterial cell. BolA is a conserved protein and is found to play a significant role in curli and fimbriae formation in *E. coli.* This study further proved that CRISPRi mediated suppression of the *bol*A gene leads to inhibition of biofilm formation through curli and fimbriae inhibition. Hence, it may be proposed as a possible target for intervention of biofilm mediated infections.

## Background

**Fig. 1:**
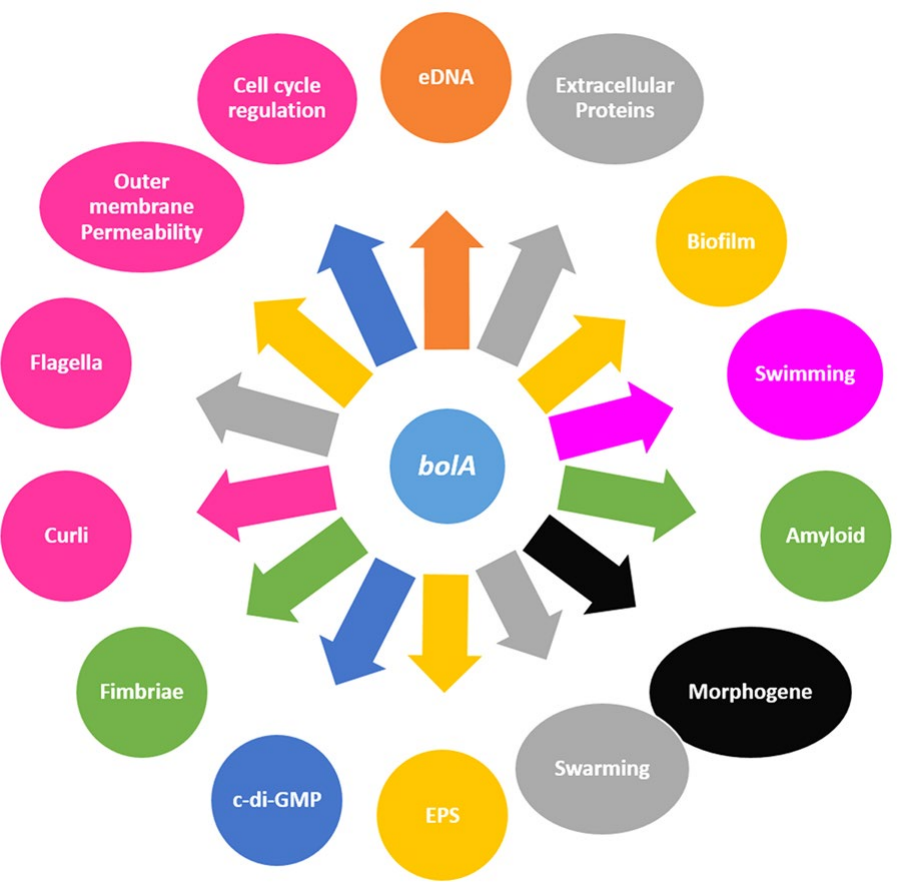
Cellular mechanisms in *E. coli* connected to the *bolA* gene/BolA protein

*Escherichia coli* BolA and its homologs established a widely conserved protein family from prokaryotes to eukaryotes, called the BolA-like protein family. Being a DNA-binding regulator, BolA promotes spherical morphology on its overexpression in *E. coli* and is one of the newly discovered stress regulator proteins [1]. The *bolA* gene has pleiotropic effects and controls a variety of phenotypes like biofilm production, biofilm regulation, bacterial morphology, fimbria-like adhesins, curli fiber formation, membrane permeability, and flagella formation (Fig. 1) [1, 2]. It has been reported that BolA is involved in the repression of flagella synthesis, and it induces the genes related to the TCA cycle [2]. Under stress conditions, the *bolA* helps in cell survival. Its overexpression leads to a short spherical morphology which decreases the surface to volume ratio, causing a reduction in the exposed surface to unfavourable environmental conditions [3].

*Escherichia coli* is the causative agent of many human infections like gastrointestinal and extraintestinal infections (ExPEC) such as septicemia, cystitis, pyelonephritis, and meningitis in newborns. Among them, urinary tract infections (UTIs) affect millions of human beings and considered to be a community-acquired infection caused by the uropathogenic *E. coli* (UPEC) [4–6]. These UPEC strains build biofilms in the form of intracellular bacterial communities (IBCs) within the superficial facet cells of the bladder or on the surface of the bladder epithelium and catheters [7], while ExPEC strains cause a wide range of human diseases varying from elementary cystitis to bloodstream infections (BSI) with mortality up to 18% within 30-days [8]. Flagella adhesins, toxins, and siderophores are associated virulence factors found with UPEC, among which α-hemolysin is a toxin, produced by around 40–50% of UPEC isolates. The α-hemolysin is a pore-forming hemolysin and its expression causes tissue damage and increases the severity of the infection [6].

Being the consequence of UPEC strains infection, bacterial biofilms are the cell aggregates enclosed in an exopolymeric substance (EPS matrix) produced by the bacteria themselves. The cells in biofilm exhibit more resistance to antimicrobial agents than in planktonic form. EPS matrix works as a diffusive barrier that prevents the penetration of antimicrobial agents to the cell and immobilizes the antibiotics. In addition to this, the biofilm diffusive barrier generates a nutrient gradient that ultimately leads to the growth rate reduction and increment in the persister cells [9, 10]. Biofilm formation in *E. coli* (e.g. UPEC) is controlled by several factors like curli, fimbriae, and auto transporter proteins such as adhesins, cellulose, and capsule as polysaccharides [7].

Among other mentioned factors, curli fibers are thin extracellular proteinaceous coiled fibers produced especially by enteric bacteria like *E. coli* and *Salmonella* spp. These fibers are aggregative and involved in cell to cell adhesion and surface attachment (both biotic and abiotic) that helps in community behaviour expression and the host colonization [11, 12]. Curli expression in bacterial cells is affected by several environmental factors like growth phase, temperature, and osmolality. In *E. coli,* curli expression is observed maximum at low temperature (22–26 °C) while at 35–37 °C its expression is found negligible [13].

Curli fibers are among the first identified functional amyloids that constitute highly stable and branchless fibers. These are 612 nm thick and work as a scaffolding agent in the formation of biofilm [14]. The formation of curli fibers occurs through a nucleation dependent self-assembly process (Type VIII secretion system) where they acquire amyloid fold as their native fold structure [15]. The CsgA is the major subunit of curli and it is capable of self-polymerizing into beta-sheet rich amyloid fibers that bind to the amyloid binding dyes like Thioflavin-T (ThT) [14].

This study was proposed to understand the role of the *bolA* gene in biofilm formation through assaying curli fibers, amyloids and fimbriae production pathways using CRISPRi as a knockdown tool. As previously known Clustered Regularly Interspaced Short Palindromic Repeats (CRISPR) is the sequence-specific RNA guided immune system in eubacteria and archaebacteria that provides a sequence-specific adaptive immunity against the foreign DNA that may be a plasmid, phage or transposons [9]. CRISPR mediated gene activation (CRISPRa for activation) or repression (CRISPRi for repression) has been derived from the type II CRISPR system of bacteria. CRISPRi and CRISPRa both utilize a deactivated or repurposed form of the Cas9 enzyme (designated as dCas9) fused with transcriptional repressors and activators, respectively [16]. The enzyme dCas9 lacks endonucleolytic activity which is achieved through the point mutation of its two domains namely RuvC-like (D10A) and HNH nuclease (H840A) domains [17].

## Methods

### Bacterial cell culture

*Escherichia coli* MG1655 was used for biofilm formation and other phenotypic studies. The bacterial cells were subcultured in Luria–Bertani (LB) broth, (Himedia labs, Mumbai, India). The cells were grown at 37 °C under shaking incubator supplemented with ampicillin 100 μg mL^−1^ and chloramphenicol 25 μg mL^−1^. Inducer anhydrotetracycline (hydrochloride, aTc) was added (2 μM) in the knockdown strains (*bol*-KD) to achieve the plasmid expressions.

### Plasmid vectors

Two plasmids were used. The first plasmid was pdCas9 for the expression of dCas9 endonuclease of *S. pyogenes* and the second one was pgRNA, for expression of sequence-specific sgRNAs. The plasmids were commercially purchased from the ADDGENE plasmids repository (Addgene no. 44249 and 44251) [17].

### Cloning of sgRNA

Two sets of primers were designed to insert the target sgRNA sequences in the vector pgRNA, to target the desired gene at different sites (Additional file 1: Table S1). The complementary target region is adjacent to PAM (Protospacer Adjacent Motif), i.e. following the 5′-CCN-3′ 20 bp sgRNA sequences containing 35 nucleotide part of the dCas9 handle which was commercially synthesized in the form of primers. Inverse PCR reactions were carried out to insert these sequences in the pgRNA plasmid. Reaction conditions were adjusted according to the protocol used by Larson et al. [17]. Both forward and reverse primers were phosphorylated. After the successful PCR, the results were verified by running a 1% agarose gel. The correct sequence bands (~ 2.5 kb) were eluted using a gel extraction kit (Invitrogen). The eluted products were freed from template strands by using the *Dpn*I enzyme and ligated by using a quick blunt end ligation kit (New England Biolabs). The ligated plasmids were transformed into the chemically competent *E. coli* Top 10 cells. The single colony is picked up and colony PCR was performed. The correct bands were eluted by using a gel extraction kit and the eluted product was sent for the sequencing with forward primer.

### Co-transformation

Plasmids from the confirmed clones were isolated and co-transformed along with the pdCas9 plasmid in the chemically competent *E. coli* MG1655 cells. Single colonies were picked up from the co-transformed clones (knockdown strains, *bol*-KD) and cultured in LB broth supplemented with ampicillin and chloramphenicol for further experiments.

### Total RNA isolation

The trizol method was used for total RNA isolation. The secondary culture of knockdown cells (bol-KD) supplemented with antibiotics and 2 μM aTc, were grown until the log phase (0.4 OD) [10]. For control, cells having pdCas9 and empty pgRNA plasmids were taken.

### RT-PCR and mRNA quantification

The isolated bacterial RNA was treated with RNase free DNase to remove any type of DNA contamination. Further, this RNA was used to proceed for the cDNA preparation by the cDNA Reverse Transcription Kits (Applied Biosystems, USA) as per the manufacturer’s instruction. The RT-PCR was performed, using SYBR green master mix along with 150 ng cDNA concentration with forward and reverse RT primers. The standard curves for respective transcripts were observed using 16 s rRNA as an endogenous control. The RT-PCR conditions were 95 °C for 10 min, 95 °C for 15 s, 60 °C for the 30 s and finally 72 °C for 30 s.

### Biofilm formation assay

The biofilm production assay was performed in “U” shaped 96 well microtiter plate. The overnight grown culture of knockdown strains was diluted 1:250 in the fresh sterile LB broth supplemented with appropriate concentration of ampicillin, chloramphenicol and aTc (2 μM) [10]. The 100 μL of this diluted culture was dispensed in each well and the plate was kept at 37 °C without shaking for 24 h. After 24 h incubation, the media was removed and the wells were washed with PBS buffer (pH 7.4) to remove the planktonic cells. After washing, the biofilm was fixed by using 37% formalin supplemented with 2% sodium acetate, and the plate was incubated at 4 °C for 4–6 h. The staining was performed by adding 200 μL of 0.1% crystal violet solution in each well and incubated at room temperature for 15–20 min, followed by washing with PBS. Bound dye was dissolved by using 100 μL of 95% absolute alcohol and kept at room temperature for 5 min at shaking. OD was read at 630 nm with the help of a microtiter plate reader (BIORAD).

### Extracellular material quantification

The extracellular material quantification was performed by the method used by Dressaire et al. [1]. Overnight cell culture was diluted with 1:250 in LB broth supplemented with an appropriate concentration of antibiotics and aTc (2 μM). 100 µL of the diluted culture was dispensed in each well of 96 well “U” shaped microtiter plates. The plate was incubated at 37 °C for 24 h without shaking. After 24 h growth, the media was removed and the biofilm of each well was dissolved in 250 µL of autoclaved ddH_2_O. The biofilm was properly dissolved in water by vortexing and centrifuged at 5000 rpm for 5 min. After centrifugation, the supernatant was collected, eDNA and protein concentration in EPS was measured by using Nanodrop (GE healthcare). For the exopolysaccharide quantification, the supernatant was mixed with 5% phenol solution (5 g phenol in 100 mL ddH_2_O). To this solution, 0.5% sulfuric acid reagent (0.5 g hydrazine sulfate in 100 mL sulfuric acid) was added, mixed by vortexing, and incubated at 4 °C for 1 h. After incubation, the microtiter plate was read in a microtiter reader at 490 nm.

### Curli production: Congo red agar plate Assay

YESCA agar (Yeast extract and casaminoacid) plates with 30 μg mL^−1^ Congo red (YESCA + Congo red) were prepared and supplemented with appropriate concentrations of antibiotics and inducer aTc [7]. The plates were streaked with an overnight culture of cells and incubated at 26 °C for 48 h.

### Amyloid production: ThT and Congo red fluorescence

The biofilm was grown in 96 well plates as mentioned earlier except the incubation was done at 26 °C for 48 h in LB media. To remove planktonic cells, the media was carefully removed and the biofilm cells were dissolved in autoclaved ddH_2_O (250 µL ddH_2_O per well of biofilm). These dissolved biofilm cells were used for the ThT and Congo red fluorescence assay. The ThT samples were prepared by using 25 μg mL^−1^ of ThT and incubated at room temperature for 2 h in the dark. Similarly, the 15 μg mL^−1^ of Congo red was added to the EPS sample and incubated in the dark at 37 °C for 3 h. For the ThT assay, 450 nm excitation wavelength and fluorescence spectra were recorded from 440 to 600 nm range. In Congo red assay, 497 nm was taken as excitation wavelength and fluorescence spectra were taken from 450 to 600 nm range.

### Toluidine blue O (TBO) EPS binding fluorescence assay

Toluidine blue O (TBO) binding fluorescence assay was performed as per the method of Misba et al. with slight modifications [26, 27]. The fluorescence assay was carried out by using the 48 h grown biofilm cells isolated for the ThT and Congo red fluorescence assay. Before performing the assay, 15 μg mL^−1^ of TBO was added to the sample and incubated at room temperature for 2 h in the dark. Fluorescence spectra were taken at 630 nm in the range of 600–700 nm.

### Cell viability

The XTT reduction/cell viability assay was carried out as reported by Zuberi et al. [10]. 0.4 mM menadione solution was prepared in acetone and filter sterilized and 1 mg mL^−1^ solution of XTT was prepared in PBS and filtered. To remove planktonic cells, the 24 h grown biofilm cells were washed with PBS. After washing 42 µL of XTT-menadione fresh mixture (20:1 volume of XTT and menadione) and 158 µL of PBS was suspended in each well and stored at 37 °C for 4 h in the dark. After incubation, the variation in the orange colour intensity in the microtiter plate was observed at 490 nm.

### Microscopic studies

#### Confocal laser scanning microscopy

Both control and *bol*-KD bacterial cells were grown in confocal dishes for 40 h at 37 °C for biofilm growth. After incubation, the planktonic cells were removed by washing the biofilm with PBS thrice. Before taking the image, the biofilm cells were stained with 0.2 μg mL^−1^ DAPI (4′, 6-Diamidine-2′-phenylindole dihydrochloride) and incubated at room temperature for 1 h. The biofilm cells were visualized under the Zeiss LSM 780 (Germany) confocal laser scanning microscope (CLSM).

#### Transmission electron microscopy (TEM)

Bacterial biofilm was grown in the 96 well “U” shaped microtiter plate in YESCA medium (10 g L^−1^ casamino acid, 1.2 g L^−1^ yeast extract) at 26 °C for 40 h. After incubation, the media was removed and the biofilm cells were dissolved in the filtered autoclaved ddH_2_O. The cells were washed once with ddH_2_O and observed under the TEM (JEM 2100, Jeol, Tokyo, Japan).

### Statistical analysis

All experiments reported were performed in triplicates. The results were assessed either by Student’s t-test or by one way ANOVA analysis (*p* values between *p *< 0.05 and *p *< 0.001 were considered statistically significant).

## Results

### Construction of sgRNA plasmid

Sequence-specific sgRNA expressing plasmids (CRISPRi) were synthesized by using inverse PCR. The PCR results were verified by 1% agarose gel electrophoresis (Additional file 1: Figure S1). These PCR products were purified from template strands through *Dpn*I digestion, ligated, and transformed in the chemically competent Top10 cells. The cloned cells were confirmed by colony PCR (Additional file 1: Figure S2) and further by DNA sequencing. The confirmed sgRNA plasmids were designated as InvF1 and InvF2.

### Suppression of *bolA* gene through CRISPRi

**Fig. 2:**
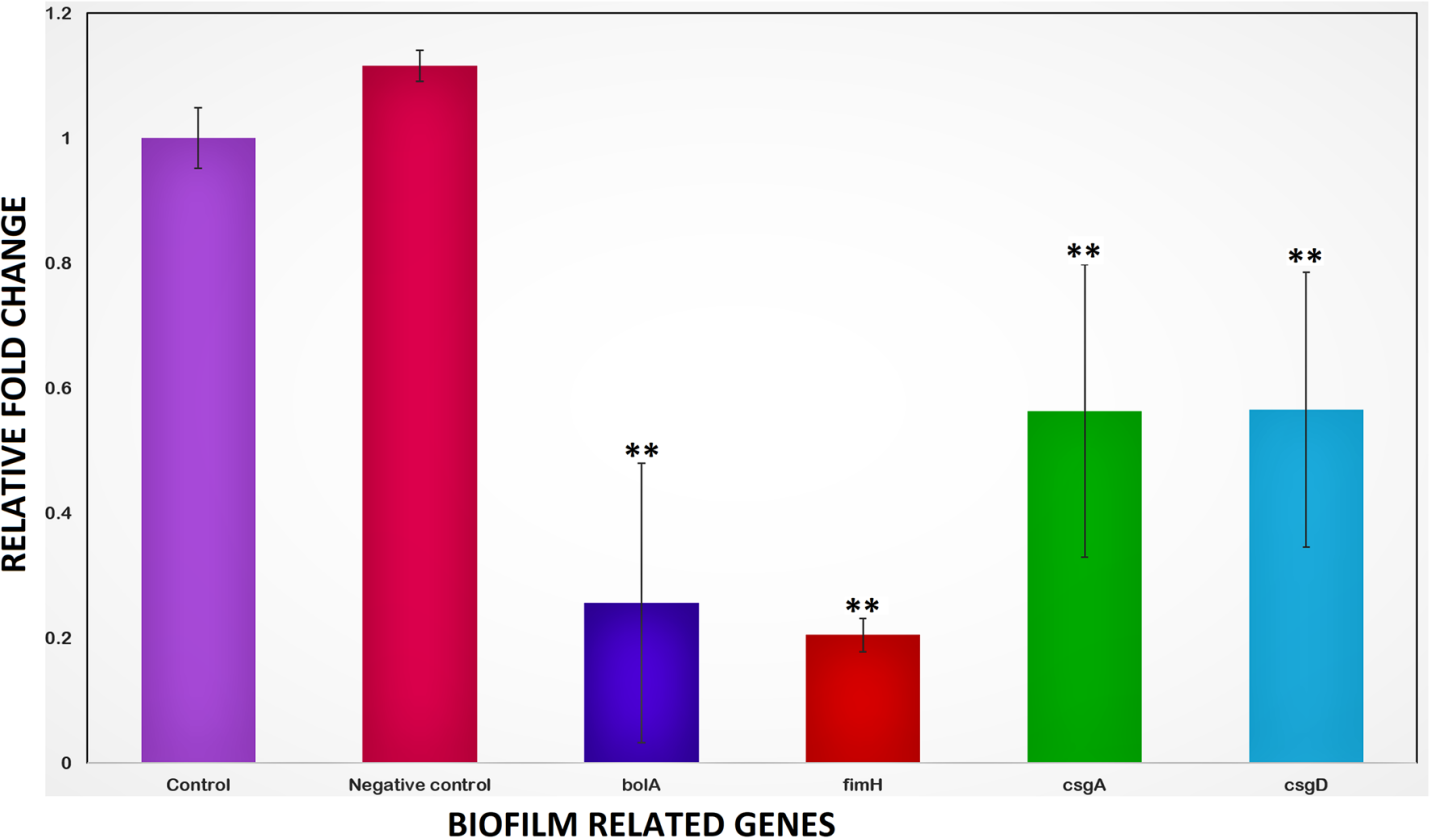
Relative qRT-PCR data of different genes (*fimH*, *csgA*, and *csgD*) in CRISPRi mediated *bolA* knocked down *E. coli* cells (*bol*-KD). The control cells consist of pdCas9 and empty pgRNA plasmids while negative control are cells without plasmid. The data represent an average of triplicate experiments

The *bolA* mRNA expression level was checked by performing real-time PCR. The RT-PCR data showed the downregulation of *bolA* gene (Fig. 2). Keeping in mind that *bolA* gene regulates the biofilm formation and controls the planktonic behavior of the cells, we have performed the RT PCR of the *bolA* gene knockdown cells (*bol*-KD) to check the mRNA levels of the curli fiber genes (*csgA* and *csgD*) and *fimH* gene of fimbriae which is an adhesive protein (FimH*).* The suppression of the *bolA* gene by 74.4% led to the suppression of curli amyloid’s major subunit *csgA* and *csgD* gene by 43.6% and 43.4%, respectively. In addition to the curli gene, the *fimH* gene was also found to be suppressed by 79.5%. This result showed that *bolA* gene expression regulates both curli and fimbriae production through some unknown mechanism. The expression data was further validated by other assays using sgRNA expressing plasmid carrying cells.

### Biofilm status of *bolA* knockdown strain

**Fig. 3:**
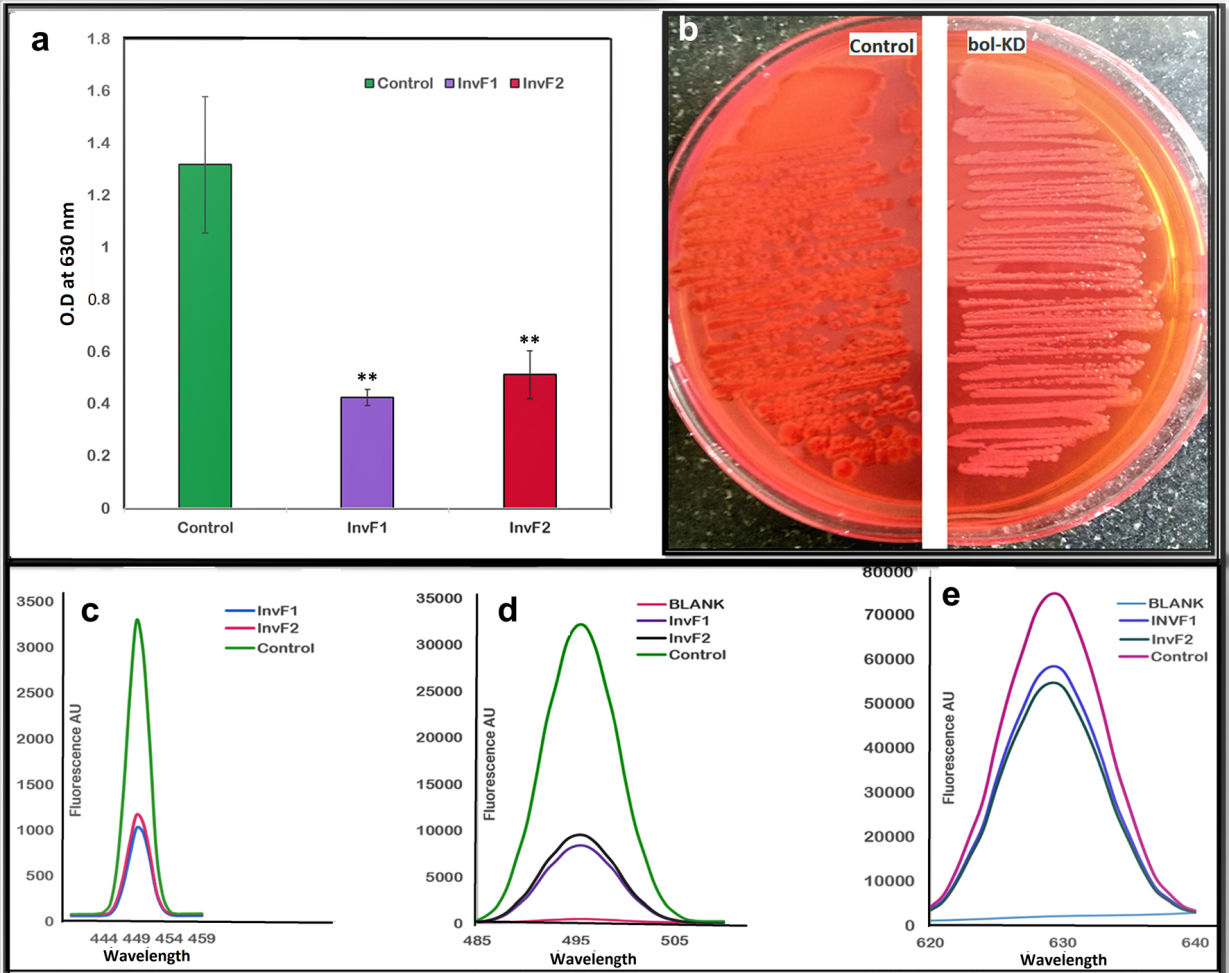
**a** Biofilm quantification with Crystal violet assay. The data represent an average of triplicate experiments ± SD (*p < 0.05, t-test, two sided) (**p < 0.005, t-test, two sided). **b** Congo red agar plates showing a whitish colony in the treated cells, an indicator of reduction in the curli production. **c** ThT fluorescence spectra. **d** Congo red fluorescence spectra. **e** TBO fluorescence spectra

The *bolA* gene is known to regulate biofilm formation. The CV assay was performed to show the biofilm formation in *bol*-KD cells and a decrease in the biofilm formation was observed. Both InvF1 and InvF2 plasmid carrying cells showed a reduction in biofilm formation by 67.8% and 61.1%, respectively (Fig. 3a), as compared to the control biofilm cells with empty sgRNA plasmid. The control cells have only empty pgRNA plasmid and pdCas9 plasmid.

### Congo red Agar plate (Curli production)

After 48 h incubation, the control cells on Congo red agar plates showed the increased production of curli fibrils, thickened the 3D texture and darker red colonies (Fig. 3b). The *bol*-KD cells showed a pale-colored or whitish colony and flat with less condensed growth as compared to the control cells.

### ThT and Congo red fluorescence

The 48 h grown *bol*-KD cells in biofilm after 2 h incubation with ThT dye showed a steep decrease in the ThT fluorescence intensity compared to the control biofilm cells (Fig. 3c). Moreover, a sharp decrease in Congo red fluorescence (Fig. 3d), intensity was also found in bol-KD cells as compared to the control biofilm cells (having empty pgRNA and pdCas9 plasmid).

### Toluidine blue O (TBO) EPS binding fluorescence

EPS TBO interaction assessment, using fluorescence spectroscopy, showed a decrease in the fluorescence intensity in *bol*-KD cells as compared to the control cells with empty pgRNA and pdCas9 plasmid (Fig. 3e).

### Extracellular material reduced in *bol*-KD cells

**Fig. 4:**
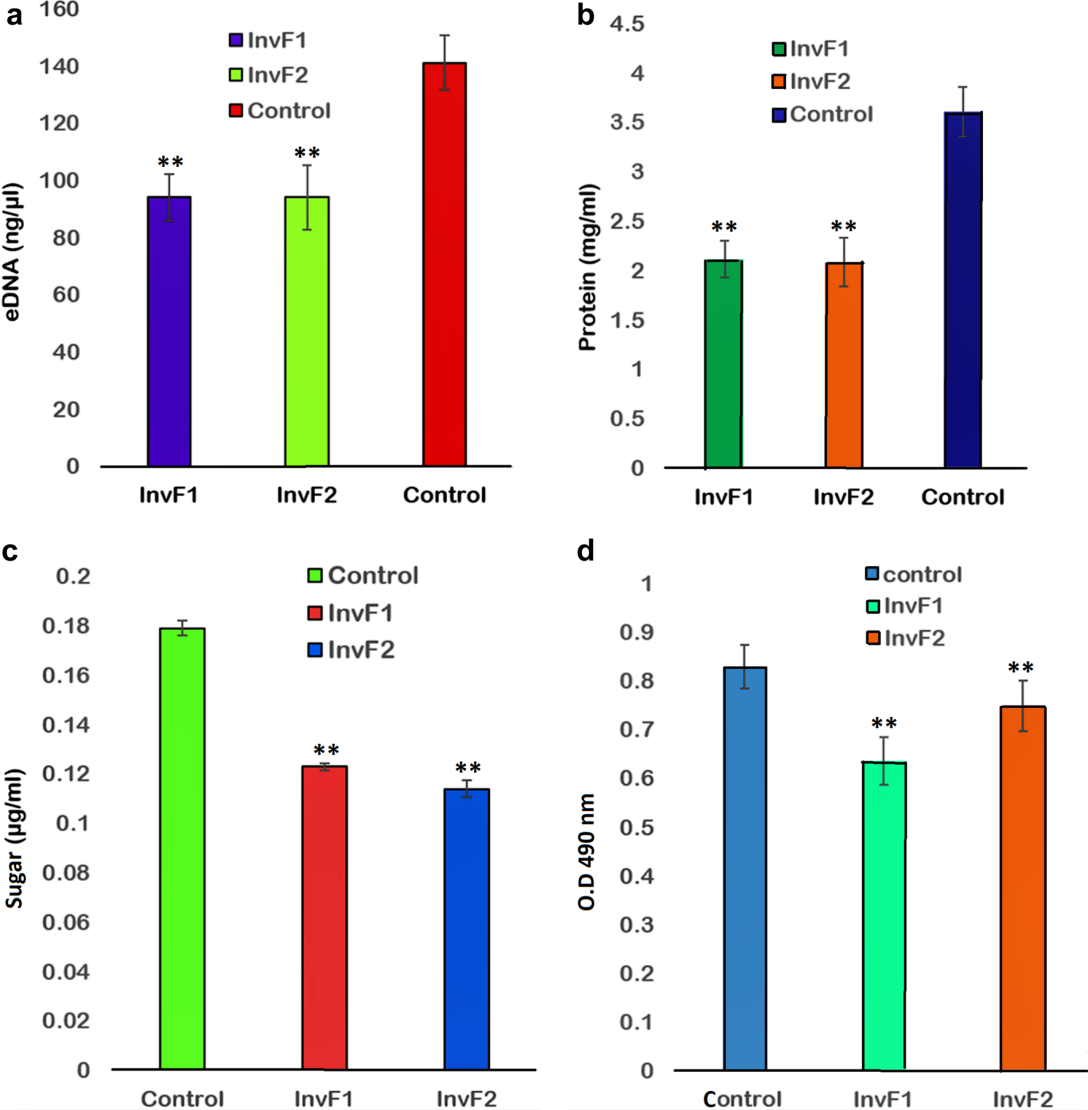
**a** eDNA estimation. **b** EPS protein estimation, **c** EPS sugar content estimation. **d** XTT reduction in cell viability assay. The *bol*-KD InvF1 cells showed 76.7% viability while the InvF2 cells showed 92% cell viability as compared to the control cells

A decrease in the extracellular DNA (eDNA), protein and sugar levels were found in extracellular material. All these components play an important role in biofilm formation. It can be seen in Fig. 4 (Fig. 4a for eDNA, Fig. 4b for protein and Fig. 4c for sugar), the eDNA, protein, and sugar levels in *bol*-KD cells (InvF1 and InvF2) showed remarkable reduction as compared to the control cells. InvF1 cells show 33.3% eDNA, 41% protein and 31.2% reduction as compared to the control. Similarly, the InvF2 cells show 33.4% eDNA, 42.2% protein and 36.3% reduction as compared to the control cells.

### Cell viability of *bol*-KD cells

The XTT reduction assay of the *bol*-KD cells revealed that both the InvF1 and InvF2 *bol*-KD cells showed 76.7% and 90.2% cell viability, respectively, as compared to the control cells (Fig. 4d). This viability data suggests that knockdown of the *bolA* gene had no considerable effect on cell viability.

### Microscopic studies

**Fig. 5:**
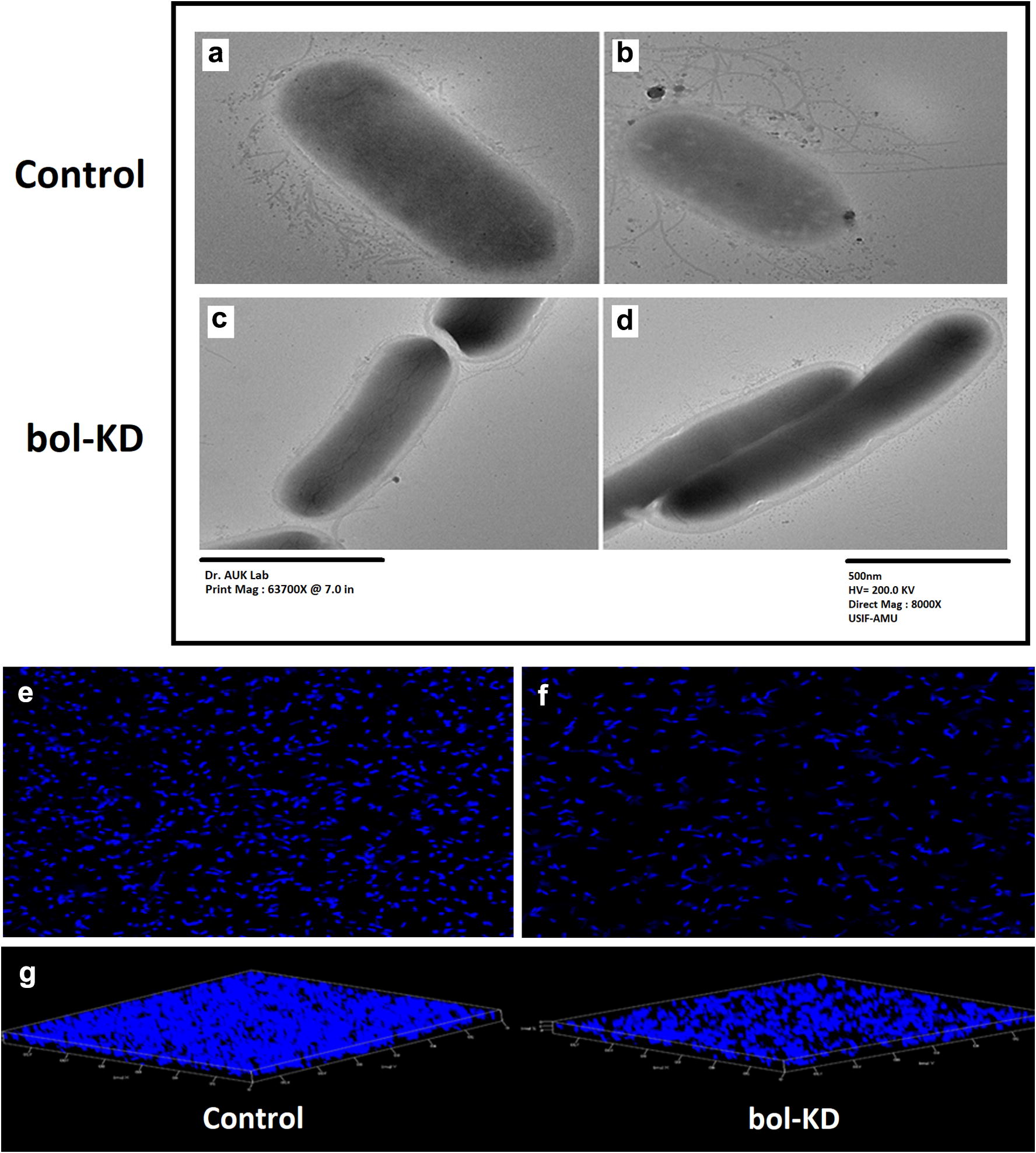
TEM images from different visual fields. **a**, **b** Control, **c**, **d** CRISPRi treated bol-KD cells. Biofilm cell adherence through CSLM (**e**–**g**). **e** Control, **f** treated bol-KD, **g** biofilm thickness and its conglomeration comparison between control and bol-KD cells

The TEM study was performed to visualize the effect of *bol*-KD on the *E. coli* biofilm cell surfaces. TEM images of the *bol*-KD biofilm cells have nearly a smooth cell surface, showing a decrease in the curli and fimbriae fibers (Fig. 5). While a cluster of curli fimbriae and other extracellular extremities can be seen in the control cells (expressing *bolA* gene).

Similarly, our CSLM results are in coherence with the TEM and other experiments results that were performed in this study. The CLSM showed a decrease in biofilm thickness as well as the cell aggregation in *bol*-KD cells while the control cells showed a thick biofilm in which the cells are more aggregated with more EPS content than in the *bol*-KD cells in biofilm (Fig. 5).

## Discussion

The biofilm formation in bacterial cells is a complex phenomenon. To understand the mechanism of change in state of bacterial cells from planktonic to sessile or biofilm, is a fascinating area of research. The biofilm formation in bacterial cells (e.g. *E. coli*) is controlled by several factors including genes, transcription regulators, two-component systems (EnvZ/OmpR and CpxA/CpxR), bacterial secondary messenger (e.g. c-di-GMP) and chemical molecules like in quorum sensing [14, 15].

The BolA protein is found to be a transcriptional switch that regulates the transition of planktonic to the biofilm stage in bacterial cells. The BolA protein has pleiotropic effects and it regulates the flagellar gene expression and biofilm formation. In addition to the BolA, a common bacterial secondary messenger c-di-GMP also regulates the bacterial cell motility, cell cycle regulation, biofilm formation and virulence. The overexpression of the bolA gene reduces the swimming motility in *E. coli* by regulating its flagellar assembly and that eventually leads an increase in the biofilm formation [1]. It is also reported that alteration in the *bolA* gene expression affects the outer membrane properties and also beta-lactamase AmpC, PBP5, PBP6 and carboxypeptidases. The overexpression of *bolA* causes a reduction in membrane permeability, that ultimately leads to a decrease in the penetration of the high molecular mass antibiotics into the cells [18]. In view of these facts, we have initiated this study to explore the role of *bolA* in the biofilm formation and expression of curli amyloid and fimbriae (*fimH*) like associated virulence factors.

Curli proteins are secreted through the type VIII secretion system (T8SS). In *E. coli*, curli synthesis involves mainly seven genes from *csgA* to *csgG*. The curli biogenesis is controlled by two different operons namely *csgBAC* and *csgDEFG*. The CsgA and CsgB are major and minor subunits, respectively, and form the structural components of the curli fiber. The CsgC is the periplasmic chaperone whereas, CsgE, CsgF and CsgG form the secretion machinery of the curli. The *csgD* gene is the master regulator of curli biogenesis, biofilm production, cellulose formation and it controls the *csgBAC* operon at the transcriptional level [15, 19]. The CsgD also controls the *adrA* which regulates cellulose biosynthesis, post-transcriptionally and encodes another common biofilm matrix component [20]. The activity of the CsgD is under the control of several regulatory systems that respond to several environmental factors like temperature, osmolality, pH and nutrient conditions. Curli fibers help bacterial cells to adhere to biotic (e.g. host proteins) and abiotic surfaces that in turn, help in the biofilm formation and pathogenicity [20].

The *bolA* gene of the *E. coli* K-12 MG1655 cells was suppressed using CRISPRi technology. The gene expression data showed the downregulation in the mRNA expression level by 74.4%. *bol*-*KD* cells were further explored for the expression of the major curli subunit *csgA* gene and the master regulator of biofilm and curli synthesis genes *csgD,* whereas, both *csgA* and *csgD* genes in *bolA* knockdown cells were found downregulated. The *csgA* and *csgD* genes show suppression by 43.6% and 43.4%, respectively. In *E. coli*, Type I fimbriae play a role in urinary tract infections that help in the adhesion to the host cells e.g. to the mannose expressing receptors on uroepithelium and promotes the intracellular bacterial communities formation. These adhesin proteins are encoded by *fim* genes, having two transcription units, working independently. These units encode a polycistronic operon that forms the structural components (FimA, FimF, FimG, and FimH), recombinase FimB and FimE and pilus assembly system (FimC and FimD) [21]. Further, we have checked the expression level of another adhesion protein FimH of fimbriae which is encoded by the *fimH* gene. The FimH is a mannose-binding protein and acts as an adhesive protein to help bacterial cells to attach on cells surfaces and it occurs at the tip of the fimbriae [22]. The gene expression data showed 79.5% suppression of *the fimH* gene in *bol*-KD cells, which is very high as compared to the suppression of curli genes (Fig. 2). It was conclude from the gene expression data that *bolA* gene is somehow affecting the expression of curli and fimbriae gene in *E. coli* biofilm formation.

The functional amyloids form a structural framework of the extracellular matrix in the biofilm. Amyloid formation is associated with several incurable neurodegenerative diseases in the human being that ranges from Parkinson’s disease (PD), Alzheimer’s disease (AD), Huntington’s disease, and prion diseases to Spinal muscular atrophy (SMA). The amyloid structure is highly stable and is generally resistant to the harsh denaturing conditions. General proteases are even, unable to degrade it [20].

In *E. coli* and *Salmonella* sp., the Congo red (CR) is frequently used to assess the curli production. The CR dye does not inhibit the growth of cells and binds to curliated whole cells and can be used to quantify the curliation in whole cells. When *E. coli* cells were grown in CR containing agar plates, there is depletion in the CR in the underlying agar of growth which suggests the curli production [22, 23]. The *bol*-KD cells were found to reduce curli amyloid formation on CR agar plates as shown in Fig. 3b. To further validate the study, we have performed the fluorescence spectroscopic assay of the biofilm cells with ThT and CR. ThT and CR dyes are the most commonly used indicator dyes that bind to curli amyloid fibrils and show increased fluorescence upon binding [24, 25]. The fluorescence spectra of both ThT and CR showed a sharp and very high fluorescence intensity in control cells while the *bol*-KD cells showed the least fluorescence intensity as compared to the control cells (Fig. 3c, d, respectively). The fluorescence data suggests that there is a reduction in the curli amyloid production in the *bol*-KD cells.

Toluidine blue O (TBO) is a cationic dye, and it is known to bind with the negative charged cell exterior EPS. In a very similar experiment to the ThT and Congo red fluorescence, we have assessed the binding of negatively charged EPS content of the biofilm cells with the cationic dye, toluidine blue oxide (TBO) with the help of fluorescence spectroscopy [26, 27]. The *bol*-KD biofilm cells show a decrease in the TBO-EPS fluorescence as compared to the control cells (Fig. 3e). This TBO fluorescence data suggest the reduction in the negatively charges extracellular content of the biofilm. But there is one difference that we have found in case of ThT and CR fluorescence spectra, that the change in fluorescence intensity is comparable between control and *bol*-KD cells in ThT and CR assay while in case of TBO the change in fluorescence spectra is negligible.

To visualize the curli and other cell surface extracellular extremities production in the biofilm cells, we have performed the TEM analysis. Our TEM data showed that the biofilm cells in the *bol*-kD cells appeared smooth with no or very low curli and fimbriae content on their cell surface (Fig. 5a–d, TEM images). The control cells in TEM images appeared to be very robust cells having thick cluster of curli, fimbriae on their surface in comparison to *bolA* knockdown cells. Further *bolA* knockdown cells appeared as elongated cells while the control cells remained spherical. We had already discussed that *bolA* is a morphogene and its overexpression causes the spherical cell morphology. The exact mechanism of how *bolA* controls the cell morphology change is not very well understood, although some reports showed that *bolA* overexpression causes a reduction in the MreB protein [28]. The MreB protein is required for the maintenance of the rod shape of the cells and its polymerization is critical for the bacterial cell cytoskeleton [28].

The crystal violet (CV) assay was also performed for the assessment of biofilm formation in *bolA* gene knockdown cells. The CV assay of *bol*-KD cells having InvF1 and InvF2 plasmids showed 67.8% and 61.1% biofilm reduction, respectively (Fig. 3a). This reduction in biofilm formation indicates the reduction in the fimbriae and curli fibers which helps in the different stages of biofilm formation [10, 14]. Further, we had confirmed the adherence and biofilm reduction with the help of a confocal microscope (CLSM) and found a decrease in the biofilm cell adherence, thickness, and cell aggregate formation in the *bol*-KD cells (Fig. 5 confocal images e-g). Both biofilm formation assay with CV and CLSM images, further validated that the *bol*-KD cells showed reduced and disintegrated biofilm.

Biofilm cells can be a thousand times more resistant to antimicrobial treatment than the planktonic cells [3]. In biofilms, the bacterial cells are surrounded with extracellular polymeric substance (EPS) and appear as microcolonies, comprised of DNA, RNA, proteins, polysaccharides, signal molecules, etc. [29]. The above-mentioned components of the EPS help to encase the cells in the biofilm. We have quantified the eDNA, extracellular protein and sugar content in the extracellular matrix of the biofilm and found a decrease in all of the above materials in the *bol*-KD cells as shown in Fig. 4a–c, respectively. We also accessed the eDNA production in the TBO agar plates, resulting a significant difference in darkness of the colonies colour in *bol*-KD and control plates, the control plate has thickened and protruded growth while the *bol*-KD cells show thin and flat growth (Additional file 1: Figure S3).

**Fig. 6:**
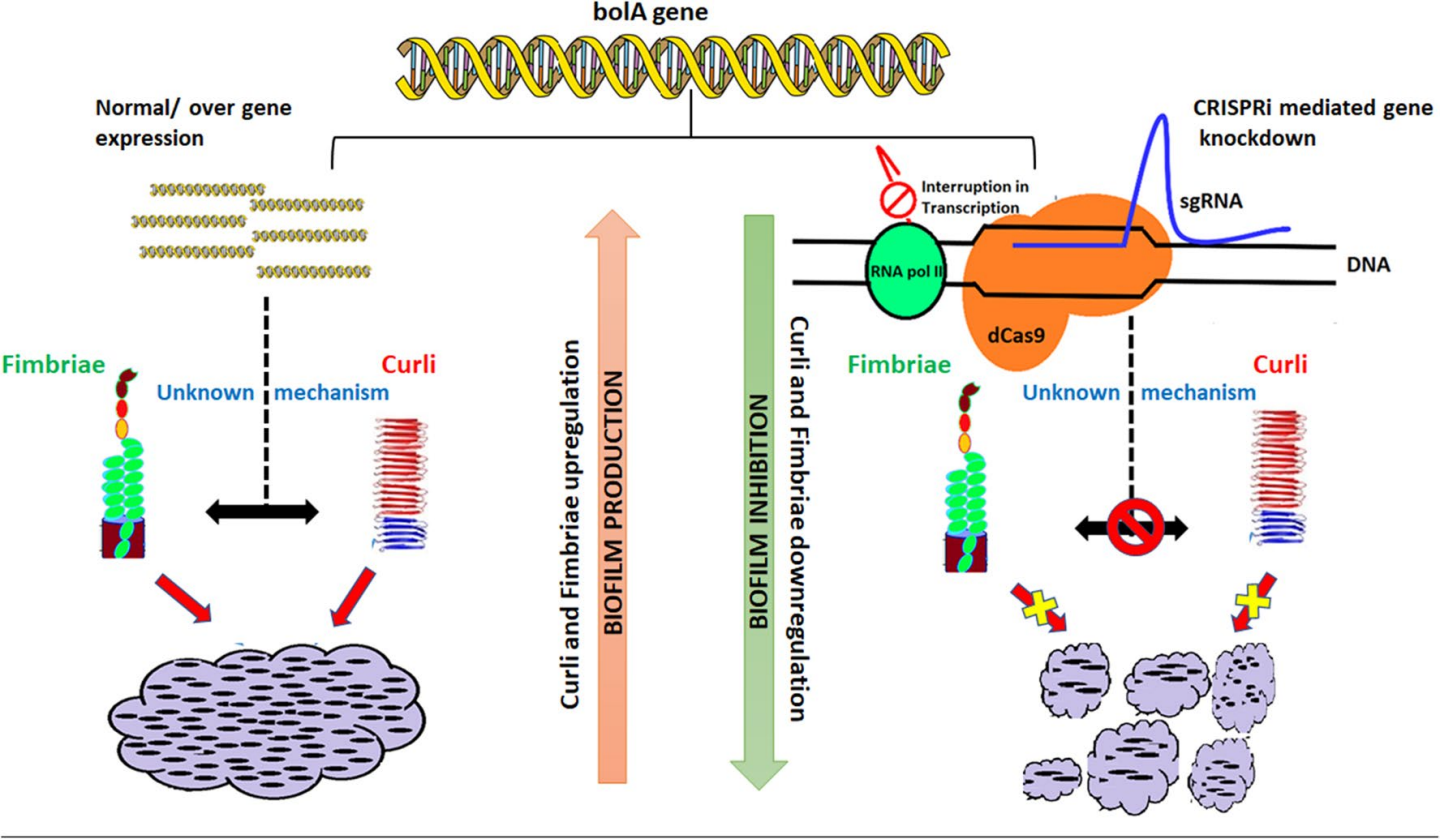
The proposed model of the study

The XTT reduction assay was performed to know whether the CRISPRi mediated knockdown of the *bolA* gene is affecting the cell viability or not (Fig. 4d)? The reduction of the tetrazolium salts (XTT) from pale or light-colored to a brightly colored product known as formazans is the basis of the cell viability assay [30]. The XTT reduction data showed that 76.7% cells of InvF1 and 92% cells of InvF2 are viable in *bolA* knockdown cells which suggests that most of the *bol*-KD cells are viable and metabolically active [31]. A model has been proposed to show the functioning of *bolA* gene downregulation which affects the curli fibers, fimbriae and biofilm formation (Fig. 6).

## Conclusion

According to our knowledge, this is the first time that is shown that the *bolA* gene plays a significant role in *E. coli* biofilm formation through regulating the *fimH*, *csgA*, and *csgD* pathways. Hence, these pathways could be the best choice to target biofilm interventions and infection control.

## Supplementary information

**Additional file 1: Figure S1.** Inverse PCR. **Figure S2.** Colony PCR of the clones with desired sgRNAs. **Figure S3.** TBO agar plates showing protruded and thick growth in control while the *bol*-KD cells show thin and flat growth. **Table S1.** List of primers used in our study.

## Data Availability

All data generated or analyzed during this study are included in this published article (and its additional information files).
