# Supplementary material for: bolA gene involved in curli amyloids and fimbriae production in E. coli: exploring pathways to inhibit biofilm and amyloid formation
Source: J Biol Res (Thessalon). 2020 Jun 17;27:10. doi: 10.1186/s40709-020-00120-7 (PMC7301969; doi:10.1186/s40709-020-00120-7)
Supplement: Supplementary file 1 — Additional file 1: Figure S1. Inverse PCR. Figure S2. Colony PCR of the clones with desired sgRNAs. Figure S3. TBO agar plates showing protruded and thick growth in control while the bol-KD cells show thin and flat growth. Table S1. List of primers used in our study. [file 40709_2020_120_MOESM1_ESM.docx]

***bolA* gene involved in curli amyloids and fimbriae production in *E. coli*: Exploring pathways to inhibit biofilm and amyloid formation**

Mohd W. Azam, Azna Zuberi and Asad U. Khan^1*^

Interdisciplinary Biotechnology Unit, Aligarh Muslim University, Aligarh (UP).

**Running title: suppresses curli amyloids and fimbriae production in *E. coli***

***Corresponding Author:** Prof. Asad U Khan, Medical Microbiology and Molecular Biology lab. Interdisciplinary Biotechnology Unit, Aligarh Muslim University, Aligarh-202002 (INDIA)

Tel: 0091-9837021912 Fax: 0091-571-2721776, (Email: [asad.k@rediffmail.com](mailto:asad.k@rediffmail.com) )

**
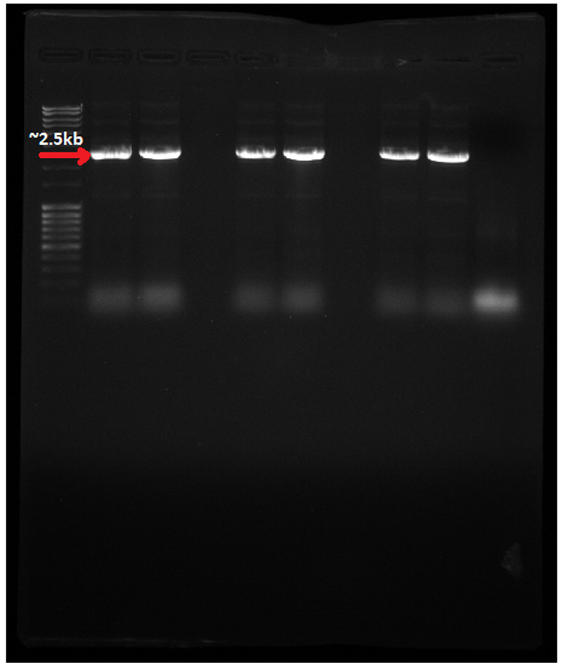
**

**Figure S1: Inverse PCR**

**
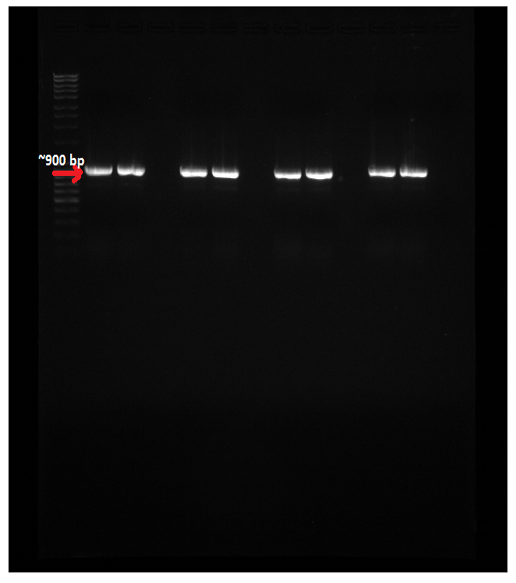
**

**Figure S2:** colony PCR of the clones with desired sgRNAs

**
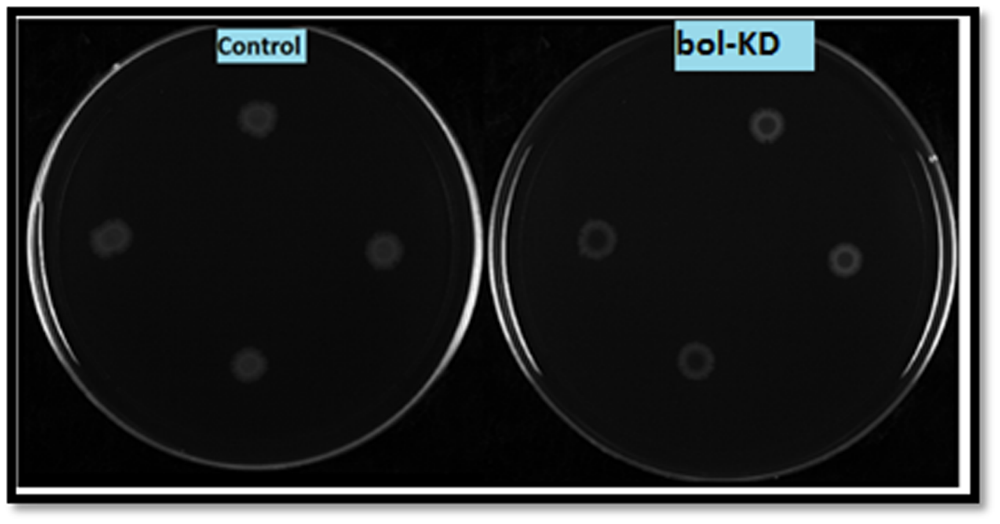
**

**Figure S3:** TBO agar plates showing protruded and thick growth in control while the *bol*-KD cells show thin and flat growth.

**Table S1**: list of primers used in our study

| **Primer name** | **Sequences** |
| --- | --- |
| **Inv-F1** | ATCCACTACTTCGAGGAATAGTTTTAGAGCTAGAAATAGCAAGTTAAAATAAGGC |
| **Inv-F2** | AGCTTTCATCCACTACTTCGGTTTTAGAGCTAGAAATAGCAAGTTAAAATAAGGC |
| **BEc-F-colony** | GGGTTATTGTCTCATGAGCGGATACATATTTG |
| **BEc-R-colony** | CGCGGCCTTTTTACGGTTC |
| **Bol-RT-F** | CCAACCCGTATTCCTCGAAGT |
| **Bol-RT-R** | GCCGGCTGGGACATTG |
| **FimH RT forward** | GATGCGGGCAACTCGATT |
| **FimH RT Reverse** | CGCCCTGTGCAGGTGAA |
| **csgA-FW** | GCGGTAATGGTGCAGATGTTG |
| **csgA-RW** | CGTTGGGTCAGATCGATTGA |
| **csgD-FW** | CGGAATCAGCCCTCCTTACTC |
| **csgD-RW** | GCGCCGATACGCAGCTTAT |
